# Postoperative radiotherapy to the neck for pN1 status HNSCC patients after neck dissection

**DOI:** 10.1038/s41598-022-17932-3

**Published:** 2022-08-11

**Authors:** Jia Wang, Xuan Su, Xing Zhang, Wenkuan Chen, Jibin Li, Zhongyuan Yang, Xiyuan Li, Jingtao Chen, Ying Zhang, Shuwei Chen, Ming Song

**Affiliations:** 1 Department of Head and Neck Surgery, Sun Yat-Sen University Cancer Center; State Key Laboratory of Oncology in South China; Collaborative Innovation Center for Cancer Medicine, Guangdong Key Laboratory of Nasopharyngeal Carcinoma Diagnosis and Therapy, 651 Dong Feng Road East, Guangzhou, 510060 China; 2 Department of Clinical Research, Sun Yat-Sen University Cancer Center; State Key Laboratory of Oncology in South China; Collaborative Innovation Center for Cancer Medicine, Guangdong Key Laboratory of Nasopharyngeal Carcinoma Diagnosis and Therapy, Guangzhou, 510060 China

**Keywords:** Cancer, Surgical oncology

## Abstract

The significance of postoperative radiotherapy (PORT) to the neck for pN1 status head and neck squamous cell carcinomas (HNSCC) after neck dissection is unclear. A total of 208 patients with pN1 status HNSCC treated from January 1, 2001, to December 31, 2014, were enrolled in the current study. The 5-year regional recurrence-free survival (RRFS), overall survival (OS) and distant metastasis-free survival (DMFS) were compared between patients with or without PORT to the dissected neck. Moreover, the stratified Cox proportional hazards models were used to assess the association between PORT to the neck and survival before and after propensity score matching. Seventy-nine patients received PORT to the neck, while 129 did not. All patients were followed for over 5 years, with a median follow-up duration of 64.6 months. The PORT group did not show better survival results than the group without PORT to the neck in RRFS, OS or DMFS. Moreover, no evidence showed that PORT to the neck was independently associated with 5-year survival. PORT to the neck for pN1 status HNSCC after neck dissection did not lead to better survival. However, it is necessary to conduct prospective randomized clinical trials to confirm these results.

## Introduction

Head and neck cancer remains a significant global health problem. Worldwide, each year, more than 830,000 individuals are diagnosed with this disease, and more than 430,000 patients die from this disease^1^; approximately 90% of these cases are HNSCCs^2^. The main treatment for HNSCCs is surgery or chemoradiotherapy. The neck is usually treated with the same modality as the primary tumor, as nodal metastasis is considered the most important prognostic factor of HNSCCs in the absence of distant metastases^3^. In patients treated surgically, neck dissection can be performed with or without postoperative radiotherapy (PORT) or concomitant chemotherapy^4^. According to the National Comprehensive Cancer Network (NCCN) guidelines^5^, patients with high risk factors, including extranodal extension, pT3-4 status, pN2-3 status, positive margins, and vascular or nerve invasion, are recommended to receive PORT after neck dissection. However, whether PORT to the neck is necessary after adequate neck lymph node dissection in pN1 patients is controversial. In February 2019, the American Society of Clinical Oncology (ASCO) commission reached an expert consensus^6^ to recommend that adjuvant neck radiotherapy should not be administered to patients with pathologically node-negative (pN0) disease or with a single pathologically positive node (pN1) after adequate neck dissection unless there are indications according to the primary tumor characteristics, such as perineural invasion, lymphovascular space invasion, or T3-4 primary cancer. However, the recommendation is based on intermediate-level evidence, and the strength of the recommendation is moderate. According to the literature, the regional control rate is not poor after adequate neck dissection^7–9^; thus, the significance of implementing PORT to the dissected neck remains unclear. Even for patients with high-risk factors, including pT3-4 status, positive margins, and vascular or nerve invasion, the necessity of postoperative treatment to improve regional control in the neck remains to be explored since local control is guaranteed by PORT performed effectively to the primary site following the NCCN guidelines.

In the present study, we explored the significance of PORT to the neck after neck lymph node dissection for pN1 HNSCC patients and aimed to provide evidence to reduce treatment-related complications and improve survival outcomes. We tried to further specify the principles of PORT and make this treatment more precise and individualized.

## Materials and methods

### Ethics approval

 The study was reviewed and approved by the ethics committee of the Sun Yat-sen University Cancer Center. All patients involved in the study consented to participate, and written informed consent was obtained.

### Study performance statement

 We confirm that all methods were performed in accordance with the relevant guidelines and regulations.

### Patient characteristics

**Figure 1:**
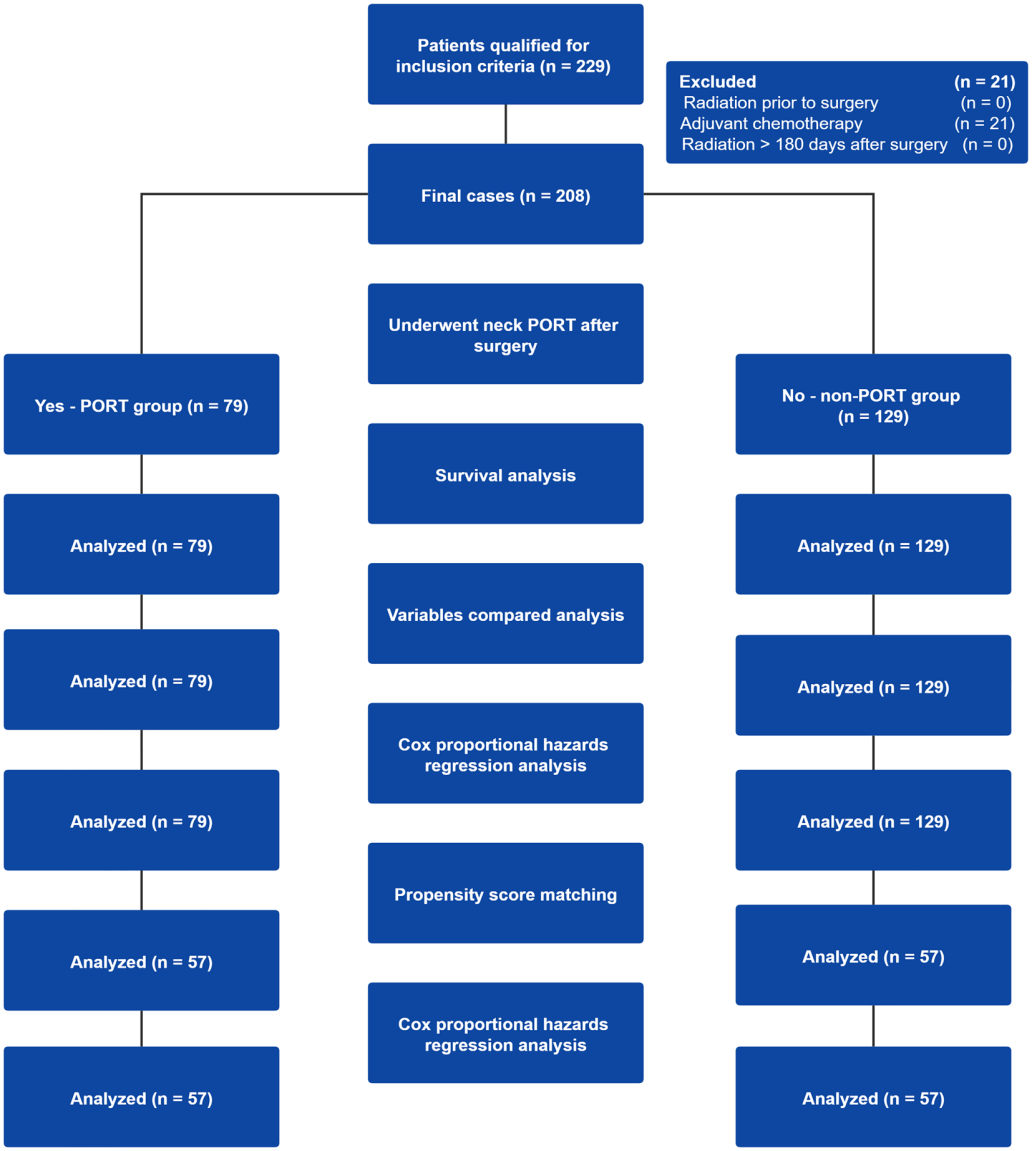
Study profile of the number of patients qualified and number analyzed in the PORT group and non-PORT group.

Consecutive patients with HNSCC,including oral, oropharyngeal (p-16 negative), and hypopharyngeal cancer, diagnosed at the Sun Yat-sen University Cancer Center from January 01, 2001 to December 31, 2014 were identified. Patients with a pathologic stage of T1-4N1M0 according to the American Joint Committee on Cancer (AJCC) 8th edition were included. Patients who received atypical treatments, including radiation before surgery, adjuvant chemotherapy, and radiation > 180 days after surgery, were excluded (Fig. 1).

All patients underwent resection of primary cancer and dissection of the ipsilateral neck with or without postoperative radiotherapy for primary cancer, according to the NCCN guidelines. Based on whether the patients underwent PORT to the neck, a PORT group and non-PORT group were created.

### Evaluation and follow-up

The regional recurrence-free survival (RRFS), overall survival (OS) and distant metastasis-free survival (DMFS) were analyzed. Five-year RRFS, OS, and DMFS rates were calculated with a 95% confidence interval (CI), and with cancer-related death or reaching the 5-year follow-up timepoint as the endpoint. All time-to-event outcomes were measured from the date of treatment to the date of the event. RRFS was calculated from the date of treatment to the date of first regional failure. OS was calculated from the date of treatment to death. DMFS was calculated from the date of treatment to the date of first remote failure. Clinical, pathologic, and epidemiologic variables were compared between the two groups.

### Statistical analysis

The Kaplan–Meier method was used to calculate the survival function, and the log-rank test was used to test for the differences between groups. The reverse Kaplan–Meier method was used to calculate the median follow-up time. The stratified Cox proportional hazards model was used to assess the association of variables with survival outcomes.

We further performed propensity score matching to control for potential selection bias in the delivery of regimens. Therefore, 1:1 matching was used for the variables given in Table 1 with a *P* ≤ 0.05 and considered a risk factor for primary cancer. The match tolerance was 0.20. Based on the matched data, outcomes were compared between the two groups using Kaplan–Meier and stratified Cox proportional hazards models.

**Table 1:** Clinical characteristics.

Classifications	PORT Group (N = 79)	non-PORT Group (N = 129)	Overall	*P*
**Age** (mean)	54.34	54.18		.921
**Sex**				
M	67 (84.8%)	92 (71.3%)	159	.029
F	12 (15.2%)	37 (28.7%)	49	
**Cigarette use**				
Yes	49 (62%)	65 (50.4%)	114	.115
No	30 (38%)	64 (49.6%)	94	
**Alcohol consumption**				
Yes	36 (45.6%)	44 (34.1%)	80	.108
No	43 (54.4%)	85 (65.9%)	128	
**pT status**				
pT1	14 (17.7%)	20 (15.5%)	34	.427
pT2	43 (54.4%)	62 (48.1%)	105	
pT3	9 (11.4%)	26 (20.2%)	35	
pT4	13 (16.5%)	21 (16.3%)	34	
**Primary site**				
Oral cavity	19 (24.1%)	114 (88.4%)	133	0.000
Oropharynx	29 (36.7%)	5 (3.9%)	34	
Hypopharynx	31 (39.2%)	10 (7.8%)	41	
**High-risk factors in the primary site**				
Perineural invasion	2 (2.5%)	0	2	.107
Lymphovascular invasion	2 (2.5%)	1 (0.8%)	3	
Positive margin	1 (1.3%)	0	1	
None	74 (93.7%)	128 (99.2%)	202	
**Lymph nodes**				
Median	16.0	18.0	NA	.164
Minimum	4	4	NA	
Maximum	57	78	NA	
**PORT to the primary bed**				
Yes	73 (69.1%)	109 (84.5%)	182	.130
No	6 (9.9%)	20 (15.5%)	26	
**Concomitant chemoradiotherapy**				
Yes	13 (16.5%)	19 (14.7%)	32	.843
No	66 (83.5%)	110 (85.3%)	176	

*M* male, *F* female, *pT status* pathologic tumor status, *NA* not applicable.

Continuous variables were compared using the Student’s *t-*test, and nonnormally distributed variables were compared using the nonparametric Mann–Whitney test. Categorical variables were compared using the Pearson χ^2^ test or Fisher’s exact test. SPSS version 26.0 (SPSS Inc) was used all statistical analyses. All *P* values were two-sided. *P* ≤ 0.05 was considered statistically significant.

### Consent for publication

The authors declare that we agree to publish this manuscript.

## Results

### Patient characteristics

A total of 208 pN1 HNSCC patients were included in the study. The average age was 54.2 years. A total of 137 patients had oral cancer, 34 patients had oropharyngeal cancer, and 41 patients had hypopharyngeal cancer. There were 159 males (76.4%) and 49 females (23.6%). Thirty-four patients had stage pT1 HNSCC, 105 patients had stage pT2 HNSCC, 35 patients had stage pT3 HNSCC, and 34 patients had stage pT4 HNSCC. Seventy-nine patients underwent PORT to the neck, while 129 patients did not. The numbers of lymph nodes removed for patients and also the median, minimum and maximum numbers in each group are presented in Table 1.

### Survival analysis

**Figure 2:**
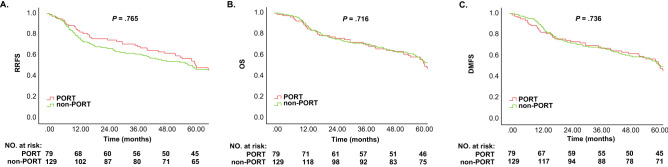
Kaplan–Meier curves for (**A**) 5-year RRFS of the two groups, (**B**) 5-year OS of the two groups, and (**C**) 5-year DMFS of the two groups.

All patients were followed for over 5 years, with a median follow-up duration of 64.6 months (95% CI 61.50–68.85). There were no differences observed between the two groups in terms of age, cigarette smoking habits, alcohol use, pT status, risk of primary cancer, PORT to the primary bed, or concomitant chemoradiotherapy. There were more males in the PORT group than in the other group (*P* = 0.029). There was a significant difference in the frequency of the primary site between groups (*P* < 0.001) (Table 1). The RRFS rates of the PORT group and non-PORT group were 54.4% (95% CI 0.43–0.66) and 50.4% (95% CI 0.42–0.59) (*P* = 0.668), respectively; the OS rates were 58.2% (95% CI 0.47–0.69) and 58.1% (95% CI 0.50–0.67) (*P* = 1.000), respectively; and the DMFS rates were 57.0% (95% CI 0.46–0.68) and 55.0% (95% CI 0.46–0.64) (*P* = 0.886), respectively. The median RRFS time was 62.0 months in the PORT group and 60.5 months in the non-PORT group (*P* = 0.765). The median OS time was 62.1 months in the PORT group and 65.3 months in the non-PORT group (*P* = 0.716). The median DMFS time was 62.0 months in the PORT group and 62.2 months in the non-PORT group (*P* = 0.736) (Table 2). The Kaplan–Meier curves are presented in Fig. 2.

**Table 2:** Survival comparison.

Classification	Groups		*P*
	PORT	Non-PORT	
**5-Year survival rate (%)**			
RRFS	54.4	50.4	.668
OS	58.2	58.1	1.000
DMFS	57.0	55.0	.886
**5-Year median survival time (months)**			
RRFS	62.0	60.5	.765
OS	62.1	65.3	.716
DMFS	62.0	62.2	.736
**5-Year survival rate of T3-4 groups (%)**			
RRFS	45.5	46.8	.916
OS	54.5	51.1	.787
DMFS	50.0	48.9	.934
**3-Year survival rate (%)**			
RRFS	70.9	62.0	.230
OS	72.2	71.3	1.000
**3-Year survival rate of T1-2 groups (%)**			
RRFS	75.0	64.4	.262

*RRFS* regional recurrence-free survival, *OS* overall survival, *DMFS* distant metastasis-free survival.

In terms of T status, the 5-year RRFS rates of patients with pT1-2 and pT3-4 disease were 57.1% (95% CI 0.44–0.71) and 47.8% (95% CI 0.26–0.70) in the PORT group (*P* = 0.468) and 52.4% (95% CI 0.41–0.64) and 46.8% (95% CI 0.32–0.62) in the non-PORT group (*P* = 0.586) (Table 3). In the separate comparison of patients with pT3-4 disease, the RRFS rates were 45.5% (95% CI 0.23–0.68) and 46.8% (95% CI 0.32–0.62) for those treated with and without PORT to the neck (*P* = 0.916). The OS rates were 54.5% (95% CI 0.32–0.77) and 51.1% (95% CI 0.36–0.66), respectively (*P* = 0.787). The DMFS was 50.0% (95% CI 0.27–0.73) and 48.9% (95% CI 0.34–0.64) (*P* = 0.934) (Table 2). Some data were analyzed over 3 years of follow up, but no significant differences observed, as presented in Table 2.

**Table 3:** Survival comparison.

Classification	pT status		*P*
	pT1-2	pT3-4	
**5-Year regional recurrence-free survival of groups (%)**			
PORT	57.1	47.8	.468
non-PORT	52.4	46.8	.586

*RRFS* regional recurrence-free survival, *pT status* pathologic tumor status.

The results of the stratified Cox proportional hazards multivariable model to assess for potential associations between each variable and survival for all 208 patients are presented in Table 4. Only age was observed to be independently associated with 5-year survival, including RRFS (hazard ratio [HR], 1.02; 95% CI 1.00–1.03; *P* = 0.041), OS (HR, 1.01; 95% CI 1.01–1.03; *P* = 0.006) and DMFS (HR, 1.01; 95% CI 1.01–1.03; *P* = 0.007). PORT to the neck was not associated with RRFS (HR, 1.26; 95% CI 0.82–1.93; *P* = 0.296), OS (HR, 1.11; 95% CI 0.72–1.69; *P* = 0.642), or DMFS (HR, 1.15; 95% CI 0.75–1.78; *P* = 0.523). After propensity score matching, a cohort with 114 patients was generated, of whom 57 received PORT to the neck, and 57 did not. None of the variables, including age and PORT to the neck, were associated with survival in the propensity-score matched cohort (Table 5). No significant difference in survival was observed from the Kaplan–Meier curves (Fig. 2 and Supplemental Figures).

**Table 4:** Cox proportional hazards regression analysis of survival in all 208 patients.

Characteristic	RRFS				OS				DMFS			
	Univariable		Multivariable		Univariable		Multivariable		Univariable		Multivariable	
	HR (95% CI)	*P*	HR (95% CI)	*P*	HR (95% CI)	*P*	HR (95% CI)	*P*	HR (95% CI)	*P*	HR (95% CI)	*P*
Age	1.01 (1.00–1.03)	.020	1.01 (1.00–1.03)	.041	1.02 (1.01–1.03)	.004	1.02 (1.01–1.03)	.006	1.02 (1.01–1.03)	.004	1.02 (1.01–1.03)	.007
Sex	0.98 (0.71–1.35)	.882	0.96 (0.63–1.47)	.863	0.96 (0.70–1.33)	.824	0.94 (0.61–1.44)	.765	1.01 (0.73–1.40)	.949	1.06 (0.69–1.62)	.800
Cigarette use	1.03 (0.78–1.36)	.840	1.24 (0.80–1.91)	.331	1.02 (0.77–1.34)	.909	1.28 (0.82–1.99)	.279	1.06 (0.81–1.40)	.667	1.35 (0.87–2.12)	.183
Alcohol consumption	0.85 (0.64–1.13)	.262	0.74 (0.50–1.11)	.150	0.84 (0.63–1.11)	.212	0.72 (0.48–1.08)	.114	0.88 (0.66–1.17)	.371	0.77 (0.51–1.15)	.766
**pT status**		.586		.694		.446		.541		.370		.517
pT1	0.80 (0.50–1.30)	.375	0.85 (0.51–1.39)	.508	0.69 (0.43–1.12)	.132	0.71 (0.43–1.17)	.180	0.71 (0.44–1.14)	.157	0.74 (0.45–1.21)	.227
pT2	0.83 (0.56–1.22)	.347	0.83 (0.55–1.24)	.362	0.80 (0.54–1.18)	.266	0.79 (0.52–1.43)	.245	0.83 (0.56–1.23)	.354	0.82 (0.55–1.24)	.351
pT3	1.02 (0.64–1.64)	.927	1.01 (0.62–1.64)	.966	0.91 (0.57–1.46)	.693	0.88 (0.55–1.43)	.614	1.02 (0.64–1.64)	.928	1.00 (0.62–1.61)	.993
pT4	NA	NA	NA	NA	NA	NA	NA	NA	NA	NA	NA	NA
**Primary site**		.387		.348		.221		.375		.144		.332
Oral cavity	0.81 (0.57–1.16)	.252	0.78 (0.47–1.28)	.323	0.79 (0.55–1.12)	.183	0.84 (0.51–1.39)	.501	0.75 (0.54–1.07)	.112	0.79 (0.48–1.30)	.353
Oropharynx	0.99 (0.63–1.56)	.959	1.15 (0.71–1.88)	.577	1.03 (0.65–1.63)	.904	1.23 (0.75–2.01)	.416	1.00 (0.63–1.59)	.992	1.19 (0.73–1.94)	.494
Hypopharynx	NA	NA	NA	NA	NA	NA	NA	NA	NA	NA	NA	NA
**High-risk factors in the primary site**		.212		.309		.090		.150		.107		.222
Perineural invasion	0.31 (0.04–2.22)	.241	0.27 (0.04–2.03)	.204	0.21 (0.03–1.51)	.120	0.18 (0.02–1.38)	.100	0.24 (0.03–1.76)	.162	0.21 (0.03–1.59)	.131
Lymphovascular invasion	0.51 (0.05–5.61)	.578	0.38 (0.03–4.46)	.441	0.40 (0.04–4.45)	.455	0.29 (0.02–3.39)	.321	0.44 (0.04–4.84)	.498	0.29 (0.03–3.48)	.332
Positive margin	0.82 (0.08–7.91)	.862	0.63 (0.06–6.63)	.698	0.62 (0.06–6.03)	.681	0.47 (0.05–4.98)	.532	0.75 (0.08–7.30)	.806	0.51 (0.05–5.43)	.575
None	NA	NA	NA	NA	NA	NA	NA	NA	NA	NA	NA	NA
**PORT to the neck**	1.04 (0.78–1.38)	.784	1.26 (0.82–1.93)	.296	0.95 (0.72–1.26)	.725	1.11 (0.72–1.69)	.642	0.95 (0.72–1.26)	.717	1.15 (0.75–1.78)	.523

*RRFS* regional recurrence-free survival, *OS* overall survival, *DMFS* distant metastasis-free survival, *pT status* pathologic tumor status, *HR* hazard ratio, *CI* confidence interval, *NA* not applicable.

**Table 5:** Cox proportional hazards regression analysis of survival in 114 matched patients.

Characteristic	RRFS				OS				DMFS			
	Univariable		Multivariable		Univariable		Multivariable		Univariable		Multivariable	
	HR (95% CI)	*P*	HR (95% CI)	*P*	HR (95% CI)	*P*	HR (95% CI)	*P*	HR (95% CI)	*P*	HR (95% CI)	*P*
Age	1.01 (1.00–1.03)	.090	1.01 (0.99–1.03)	.177	1.02 (1.00–1.03)	.056	1.02 (1.00–1.04)	.077	1.01 (1.00–1.03)	.095	1.02 (1.00–1.04)	.133
Sex	0.81 (0.51–1.29)	.372	0.65 (0.35–1.21)	.172	1.18 (0.72–1.54)	.780	0.66 (0.35–1.24)	.198	0.88 (0.55–1.39)	.575	0.71 (0.38–1.32)	.279
Cigarette use	1.10 (0.76–1.61)	.606	1.57 (0.85–2.91)	.149	1.06 (0.77–1.34)	.909	1.65 (0.87–3.12)	.123	1.12 (0.77–1.63)	.594	1.77 (0.94–3.33)	.077
Alcohol consumption	0.83 (0.57–1.21)	.331	0.61 (0.35–1.08)	.091	0.77 (0.53–1.12)	.177	0.55 (0.31–0.99)	.045	0.81 (0.56–1.18)	.273	0.57 (0.32–1.01)	.056
**pT status**		.816		.730		.790		.570		.717		.613
pT1	0.77 (0.40–1.47)	.421	0.69 (0.35–1.37)	.292	0.72 (0.38–1.38)	.320	0.61 (0.31–1.22)	.165	0.73 (0.38–1.39)	.337	0.64 (0.32–1.26)	.196
pT2	0.86 (0.51–1.47)	.588	0.75 (0.43–1.33)	.327	0.85 (0.50–1.45)	.550	0.73 (0.41–1.29)	.274	0.88 (0.52–1.49)	.628	0.76 (0.43–1.35)	.347
pT3	1.02 (0.50–2.09)	.955	0.85 (0.39–1.82)	.666	0.91 (0.45–1.87)	.802	0.78 (0.36–1.69)	.533	1.05 (0.51–2.15)	.893	0.87 (0.41–1.87)	.728
pT4	NA	NA	NA	NA	NA	NA	NA	NA	NA	NA	NA	NA
**Primary site**		.202		.076		.105		.072		.078		.060
Oral cavity	0.83 (0.51–1.35)	.451	0.75 (0.40–1.40)	.359	0.84 (0.51–1.36)	.466	0.87 (0.45–1.59)	.605	0.78 (0.48–1.27)	.322	0.77 (0.41–1.44)	.407
Oropharynx	1.25 (0.72–2.17)	.431	1.55 (0.84–2.86)	.164	1.36 (0.78–2.37)	.273	1.70 (0.92–3.17)	.093	1.31 (0.75–2.28)	.339	1.64 (0.89–3.04)	.115
Hypopharynx	NA	NA	NA	NA	NA	NA	NA	NA	NA	NA	NA	NA
**Risk of primary**		.417		.485		.185		.247		.265		.362
Perineural invasion	0.28 (0.04–2.03)	.206	0.22 (0.03–1.76)	.155	0.18 (0.02–1.32)	.091	0.14 (0.02–1.16)	.068	0.22 (0.03–1.62)	.137	0.18 (0.02–1.44)	.105
Lymphovascular invasion	0.47 (0.04–5.27)	.541	0.33 (0.03–4.24)	.396	0.35 (0.03–3.98)	.399	0.24 (0.02–3.09)	.271	0.41 (0.04–4.61)	.470	0.29 (0.02–3.70)	.339
Positive margin	0.53 (0.05–5.96)	.609	0.30 (0.02–3.97)	.58	0.41 (0.04–4.59)	.465	0.23 (0.02–3.09)	.266	0.49 (0.04–5.45)	.558	0.25 (0.02–3.41)	.300
None	NA	NA	NA	NA	NA	NA	NA	NA	NA	NA	NA	NA
**Neck PORT**	0.91 (0.63–1.32)	.620	1.01 (0.62–1.63)	.979	0.91 (0.63–1.32)	.620	1.01 (0.62–1.63)	.979	0.91 (0.63–1.32)	.613	1.07 (0.65–1.77)	.779

*RRFS* regional recurrence-free survival, *OS* overall survival, *DMFS* distant metastasis-free survival, *pT status* pathologic tumor status, *HR* hazard ratio, *CI* confidence interval, *NA* not applicable.

## Discussion

The significance of PORT to the neck for pN1 HNSCC has been controversial. The present study evaluated the benefit of PORT to the neck for pN1 HNSCCs. Because the most direct and objective outcome associated with PORT to the neck is RRFS, it was selected as one endpoint. OS and DMFS were used as other endpoints because regional progression increases the risk of distant metastasis and affects OS^10,11^.

To the best of our knowledge, no similar studies have shown evidence of whether PORT to the neck is necessary after neck dissection for pN1 HNSCC. A retrospective study^12^ reported a significant difference in the 5-year disease-free survival (DFS) rates between pN1 HNSCC patients with and without PORT after neck dissection. That study included 59 patients with T1-2/N0-1 tongue squamous cell carcinoma confirmed by pathology from 1980 to 2002, of whom 28 with stage N1 cancer received PORT, and 31 patients with stage N0 cancer did not. The results showed that the 5-year DFS rates for patients with and without PORT were 81.2% and 53% (*P* = 0.03), and the OS rates were 77% and 70.5% (*P* = 0.36), respectively. Therefore, the patients seemed to benefit from PORT after neck dissection in terms of DFS. However, this PORT benefit in DFS could come from either the primary site or dissected neck, and thus did not prove that PORT to the dissected neck alone was necessary. In addition, the results showed no significant difference in OS. If local control of the primary site is ensured, it remains unclear whether radiotherapy to the neck is necessary. The OS rate obtained in this retrospective study was similar to our rate, but the data were significantly better than ours. The reason may be because the patients included in the previous study were limited to those with tongue cancer alone, and their overall prognosis was significantly better than that of patients with oropharyngeal, hypopharyngeal and other head and neck squamous carcinomas^13,14^. However, due to the small sample size of the study and the potential selection bias, its conclusion is limited.

Our study included 69 patients with stage pT3-4 cancer, accounting for 33.2% of all participants. According to the literature, patients with advanced T-stage cancer are more likely to experience failure in terms of local control and have a worse prognosis^15,16^. If the primary site of pT3-4 cancer was treated with radiotherapy after surgery to improve the local control rate and reduce distant metastasis, the effect on the lymphatic drainage areas after neck dissection Remains unclear. Subgroup analysis showed that the 5-year RRFS rates were not significantly different between pT1-2 and pT3-4 cancer in the PORT group (57.1% vs. 47.8%, *P* = 0.468), with similar results in the non-PORT group (52.4% vs. 46.8%, *P* = 0.586), suggesting that pT1-2 and pT3-4 cancer were not significantly different in terms of regional control of the neck, with pT3-4 patients undergoing PORT to the primary site, regardless of whether PORT was performed to the neck after neck dissection. Thus, the clinical significance of PORT to the neck after neck dissection in pT3-4N1 patients need to be further explored. Analysis was performed to compare the 5-year RRFS, OS, and DMFS rates of pT3-4 patients between these two treatment groups. The results showed no differences in survival, including RRFS, OS, and DMFS (Table 2). These data suggest that PORT to the neck after neck dissection did not provide better regional control or OS after neck dissection in stage pT3-4N1 patients.

At present, the 5-year OS rate of HNSCC is less than 50%^10,17,18^ in the comprehensive treatment model. Our study showed relatively high OS rates (58.1% and 58.2%), which might be due to the relatively early N status of the enrolled patients. As seen from the Kaplan–Meier curves of RRFS in this study, although there was no significant difference in the 3-year RRFS rate between the two groups, there was a trend towards a difference (70.9% vs. 62.0%, *P* = 0.230) (Table 2). To exclude regional failure caused by the inclusion of pT3-4 cancer, we analyzed the 3-year RRFS rates of pT1-2 patients in the two groups; the rates were 75.0% and 64.6% in the PORT and non-PORT groups (*P* = 0.262) (Table 2), respectively, with no significant difference. It is possible that there could be a significant difference in 3-year RRFS between the two groups if the sample size was increased; however, there was also no significant difference in the 3-year OS rate (72.2% vs. 71.3%, *P* = 1.000) (Table 2). The results indicate that the PORT group might achieve better regional control in the short term, but there were no significant benefits in OS. Over time, there were no obvious advantages in regional control.

The Kaplan–Meier curves of RRFS in this study were approximately parallel, demonstrating that the proportional hazards assumption was established. Thus, a stratified Cox proportional hazards model was performed in the present study to assess the association between clinical profiles and survival. Both univariable and multivariable models showed no significant evidence of an association between PORT to the neck and survival, including RRFS, OS, and DMFS. Only age was independently associated with survival (Table 4). As Table 1 shows, there were differences in variables such as sex and primary site between the two groups. Although patients with high-risk factors in the primary tumors, such as perineural invasion, lymphovascular invasion, and positive margins, probably received the corresponding PORT regimen to the primary tumor, the risk factors for primary tumors was listed as a predictor as well as sex and primary site, as shown in Table 1 with *P* < 0.05, to control for the potential selection bias arising from propensity score matching that could influence survival in some way. Stratified Cox proportional hazard model data from the propensity-score matched cohort showed no significant differences, indicating that PORT to the neck and other variables had no association with survival in this study.

According to the literature^19–21^, adequate neck dissection is defined as the removal of ≥ 18 lymph nodes in the ASCO guidelines^6^. As presented in Table 1, the quality of our neck dissection was less than adequate. This was acceptable considering that the treatments were performed from year of 2001 to 2014, while the concept of adequate dissection of head and neck cancer has not yet been established. With improved neck dissection quality and locoregional control, it is worth considering whether to conduct PORT in dissected necks.

The decision why some patients received PORT to the dissected neck but some do not remains unclear. Thus far, no guidelines or consensuses have specific suggestions regarding the need for PORT to the neck after adequate neck dissection for pN1 HNSCC. In our daily diagnosis and treatment of such patients, we clearly inform patients and their families that the two options are to accept PORT to the neck or not, and that both of these options are acceptable according to current guidelines. For patients with high-risks factors in the primary sites, such as pT3-4 stage, positive margins, and vascular or nerve invasion, we would also provide these two options. Thus the patients and their families could decide whether to undergo PORT to the neck. This approach led to the fact that some patients received PORT to the neck and some did not.

Several limitations need to be addressed in this study. First, due to the retrospective nature of this study, some selection bias might occur when the adjuvant treatment strategy was made based on the experience of the attending professor. Second, as a study concerning with HNSCC, patients with laryngeal carcinoma and human papillomavirus (HPV)-positive oropharyngeal carcinoma were not enrolled, seemingly resulting in another contributor for selection bias. However, glottic laryngeal carcinoma and HPV-positive oropharyngeal carcinoma has drastically different survival outcomes to other HNSCCs. Moreover, the treatment strategies for laryngeal carcinoma and HPV-positive oropharyngeal carcinoma, including TNM stage rules and adjuvant principles, are different from those for other types of HNSCCs. Unlike other types of HNSCCs, the definition of pN1 in HPV-positive oropharyngeal cancer only depends on the number of metastatic lymph nodes. Moreover, for glottic laryngeal cancer, adjuvant cervical radiotherapy is unnecessary due to the improbability of cervical lymph node metastasis for local early lesions, such as T1 and selected T2 cases. Therefore, it was difficult to obtain a unified and high-quality enrollment criteria if these diseases were included. Last, we included more patients with T1-2 disease than patients with T3-4 disease, which might lead to a better survival outcomes. However, patients with early T stage disease are less likely to have ipsilateral lymph node metastasis. Hence, based on the pN1 enrollment criteria, it was reasonable that many more T1-2 status patients would be enrolled.

This study intended to investigate the significance of PORT to the neck after neck dissection for pN1 HNSCC patients and to explore whether PORT to the neck could be eliminated for these kinds of patients to reduce complications and improve the quality of life without affecting the treatment efficacy and survival of patients. First, the main adverse effects of radiotherapy for head and neck tumors include not only acute radiation-induced mucositis and dermatitis, which affect overall treatment tolerance but also radiation-induced xerostomia and fibrosis of the muscles, in turn affecting patient quality of life. In addition, according to literature reports, nearly 50%-65% of head and neck cancers have a risk of recurrence and metastasis^11,22^. As one of the main treatments for recurrent or metastatic HNSCCs, salvage surgery is also deeply affected by fibrosis of head and neck tissues and related anatomical structural changes after radiotherapy. Furthermore, radiotherapy can lead to secondary malignant tumors. Finally, the cost of radiotherapy is generally high; for pN1 patients with a low risk of neck recurrence, the cost-effectiveness ratio of PORT to the neck after adequate neck dissection should also be considered. Due to the limited number of cases, this study needs to be further verified by prospective, large-sample, multicenter randomized clinical trials.

## Conclusions

PORT to the neck after neck dissection for pN1 HNSCC did not lead to better survival. However, it is necessary to conduct high-quality prospective randomized clinical trials to confirm these results.

## Supplementary Information

Supplementary Figures.

Supplementary Tables.

## Data Availability

All data generated or analyzed during this study are included in this published article.

## References

[1] Bray F, Ferlay J, Soerjomataram I (2018). Global cancer statistics 2018: GLOBOCAN estimates of incidence and mortality worldwide for 36 cancers in 185 countries. CA Cancer J. Clin..

[2] Wyss A, Hashibe M, Chuang SC (2013). Cigarette, cigar, and pipe smoking and the risk of head and neck cancers: Pooled analysis in the International Head and Neck Cancer Epidemiology Consortium. Am. J. Epidemiol..

[3] Mamelle G, Pampurik J, Luboinski B (1994). Lymph node prognostic factors in head and neck squamous cell carcinomas. Am. J. Surg..

[4] Strojan P, Ferlito A, Langendijk JA (2012). Indications for radiotherapy after neck dissection. Head Neck.

[5] NCCN. The NCCN head and neck cancers clinical practice guidelines in oncology (version 3.2021)[EB/OL]. Fort Washington: NCCN, 2021[2021-04-27]. head-and-neck.pdf (nccn.org). https://www.nccn.org/professionals/physician_gls/pdf/head-and-neck.pdf

[6] Koyfman SA, Ismaila N, Crook D (2019). Management of the neck in squamous cell carcinoma of the oral cavity and oropharynx: ASCO clinical practice guideline. J. Clin. Oncol..

[7] Schmitz S, Machiels JP, Weynand B (2009). Results of selective neck dissection in the primary management of head and neck squamous cell carcinoma. Eur. Arch. Otorhinolaryngol..

[8] Wolfensberger M, Zbaeren P, Dulguerov P (2001). Surgical treatment of early oral carcinoma-results of a prospective controlled multicenter study. Head Neck.

[9] Shah JP, Candela FC, Poddar AK (1990). The patterns of cervical lymph node metastases from squamous carcinoma of the oral cavity. Cancer.

[10] Leemans CR, Braakhuis BJ, Brakenhoff RH (2011). The molecular biology of head and neck cancer. Nat. Rev. Cancer.

[11] Chow L (2020). Head and neck cancer. N. Engl. J. Med..

[12] Chen TC, Wang CT, Ko JY (2010). Postoperative radiotherapy for primary early oral tongue cancer with pathologic N1 neck. Head Neck.

[13] Scully C, Bagan J (2009). Oral squamous cell carcinoma overview. Oral Oncol..

[14] Chinn SB, Myers JN (2015). Oral cavity carcinoma: Current management, controversies, and future directions. J. Clin. Oncol..

[15] Braakhuis BJ, Brakenhoff RH, Leemans CR (2012). Treatment choice for locally advanced head and neck cancers on the basis of risk factors: Biological risk factors. Ann. Oncol..

[16] Zenga J, Wilson M, Adkins DR (2015). Treatment outcomes for T4 oropharyngeal squamous cell carcinoma. JAMA Otolaryngol. Head Neck Surg..

[17] Ho AS, Kraus DH, Ganly I (2014). Decision making in the management of recurrent head and neck cancer. Head Neck.

[18] Tobias JS, Monson K, Gupta N (2010). Chemoradiotherapy for locally advanced head and neck cancer: 10-Year follow-up of the UK Head and Neck (UKHAN1) trial. Lancet Oncol..

[19] Ebrahimi A, Zhang WJ, Gao K (2011). Nodal yield and survival in oral squamous cancer: Defining the standard of care. Cancer.

[20] Ebrahimi A, Clark JR, Amit M (2014). Minimum nodal yield in oral squamous cell carcinoma: Defining the standard of care in a multicenter international pooled validation study. Ann. Surg. Oncol..

[21] Ho AS, Kim S, Tighiouart M (2017). Metastatic lymph node burden and survival in oral cavity cancer. J. Clin. Oncol..

[22] Argiris A, Karamouzis MV, Raben D (2008). Head and neck cancer. Lancet.

