# Supplementary material for: Postoperative radiotherapy to the neck for pN1 status HNSCC patients after neck dissection
Source: Sci Rep. 2022 Aug 11;12:13696. doi: 10.1038/s41598-022-17932-3 (PMC9372140; doi:10.1038/s41598-022-17932-3)
Supplement: Supplementary file 1 — Supplementary Figures. [file 41598_2022_17932_MOESM1_ESM.pdf]

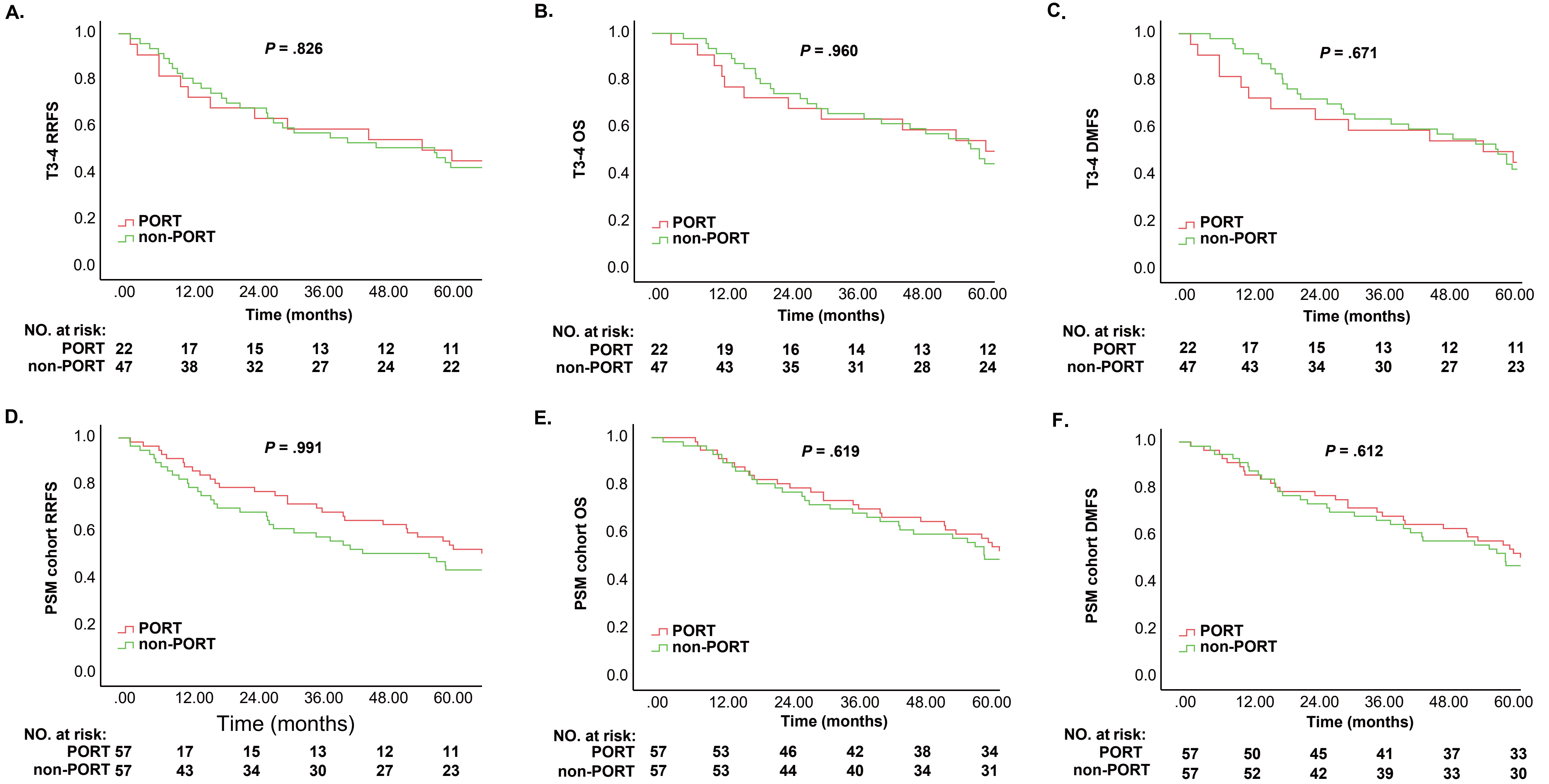

Supplemental figures. Kaplan–Meier curves for (A) 5-year RRFS of pT3-4 in the two groups, (B) 5-year OS of pT3-4 in the two groups, (C) 5-year DMFS of pT3-4 in the two groups, (D) 5-year RRFS of propensity-score matched (PSM) cohort, (E) 5-year OS of PSM cohort, (F) 5-year DMFS of PSM cohort.
