# Supplementary material for: Postoperative radiotherapy to the neck for pN1 status HNSCC patients after neck dissection
Source: Sci Rep. 2022 Aug 11;12:13696. doi: 10.1038/s41598-022-17932-3 (PMC9372140; doi:10.1038/s41598-022-17932-3)
Supplement: Supplementary file 2 — Supplementary Tables. [file 41598_2022_17932_MOESM2_ESM.pdf]

Supplemental table. Locoregional recurrence and distant metastasis data

**Classification**	Locoregional recurrence No.				Distant metastasis No.			
	PORT Group (N=79)	non-PORT Group (N=129)	Overall	*P*	PORT Group (N=79)	non-PORT Group (N=129)	Overall	*P*
**Locoregional recurrence or distant metastasis during follow-up**								
Yes	11 (13.9%)	32 (24.8%)	43	.077	2 (2.5%)	7 (5.4%)	9	.488
No	68 (86.1%)	97 (75.2%)	165		77 (97.5%)	122 (94.6%)	199	
